# Mild cognitive impairment prediction and cognitive score regression in the elderly using EEG topological data analysis and machine learning with awareness assessed in affective reminiscent paradigm

**DOI:** 10.3389/fnagi.2023.1294139

**Published:** 2024-01-04

**Authors:** Tomasz M. Rutkowski, Tomasz Komendziński, Mihoko Otake-Matsuura

**Affiliations:** ^1^RIKEN Center for Advanced Intelligence Project, Tokyo, Japan; ^2^Graduate School of Education, The University of Tokyo, Tokyo, Japan; ^3^Department of Cognitive Science, Institute of Information and Communication Research, Nicolaus Copernicus University, Toruń, Poland

**Keywords:** EEG, biomarker, mild cognitive impairment (MCI), machine learning (ML), prevention, topological data analysis (TDA)

## Abstract

**Introduction:**

The main objective of this study is to evaluate working memory and determine EEG biomarkers that can assist in the field of health neuroscience. Our ultimate goal is to utilize this approach to predict the early signs of mild cognitive impairment (MCI) in healthy elderly individuals, which could potentially lead to dementia. The advancements in health neuroscience research have revealed that affective reminiscence stimulation is an effective method for developing EEG-based neuro-biomarkers that can detect the signs of MCI.

**Methods:**

We use topological data analysis (TDA) on multivariate EEG data to extract features that can be used for unsupervised clustering, subsequent machine learning-based classification, and cognitive score regression. We perform EEG experiments to evaluate conscious awareness in affective reminiscent photography settings.

**Results:**

We use EEG and interior photography to distinguish between healthy cognitive aging and MCI. Our clustering UMAP and random forest application accurately predict MCI stage and MoCA scores.

**Discussion:**

Our team has successfully implemented TDA feature extraction, MCI classification, and an initial regression of MoCA scores. However, our study has certain limitations due to a small sample size of only 23 participants and an unbalanced class distribution. To enhance the accuracy and validity of our results, future research should focus on expanding the sample size, ensuring gender balance, and extending the study to a cross-cultural context.

## 1 Introduction

Dementia is a prevalent condition that refers to the cognitive decline experienced by older individuals, which can significantly impact their daily lives and overall wellbeing (Sperling et al., 2019). It can be caused by various factors, including Alzheimer's disease, vascular dementia, Lewy body dementia, frontotemporal dementia, and traumatic brain injuries (Livingston et al., 2020). Dementia poses a significant public health challenge worldwide, with social and economic ramifications (WHO, 2019). To assess the risk of dementia, cognitive impairment tests, such as mild cognitive impairment (MCI) prediction, are often administered (Nara et al., 2018). These tests are usually paper and pencil-based and help identify cognitive decline that is more severe than expected for a person's age but does not yet meet the clinical dementia diagnosis (Petersen et al., 1999).

Recent studies have shown that health neuroscience and EEG neuro-biomarker research are crucial in developing effective strategies for preventing dementia (Sperling et al., 2019; Rutkowski et al., 2023a). Among the promising interventions that have gained attention are reminiscent and affective stimulation, potentially boosting brain health and cognitive resilience (Hsieh and Wang, 2003; Tam et al., 2021). Dementia can affect working memory, making it difficult to encode and retrieve new memories, especially recent events or information (Brai et al., 2021). Healthy working memory can support attention switching between tasks, enhancing cognitive reserve and brain plasticity, and even protecting against dementia (Brai et al., 2021). In a study by Soto and Silvanto (2016), it was found that being consciously aware can assist in selecting and retaining important information in working memory and filtering out unimportant or distracting information in individuals in the early stages of dementia. Additionally, conscious awareness can safeguard against dementia by indicating the underlying health or impairment of the brain (Clare, 2002; Soto and Silvanto, 2016). The hippocampus is central for mood and memory; adult neurogenesis can provide hope not only for depression but also for dementia and Alzheimer's disease (Berger et al., 2020). Bar (2022) argues that helping individuals with depression or dementia regain proper neurogenesis by renewing their ability for broad associative mind-wandering. The employment of reminiscent stimulation is a therapeutic technique that employs sensory cues such as music, storytelling, or photographs to evoke memories and emotions from an individual's past. This method capitalizes on the cognitive and emotional benefits of reminiscence, which can facilitate the development of a sense of identity and connection with one's history (Pinquart and Forstmeier, 2012; Woods et al., 2018). Affective stimulation refers to activities and experiences that evoke positive emotions, reduce stress, and improve overall emotional wellbeing. This method acknowledges the relationship between emotional health and cognitive function to foster a supportive environment for brain health (Hsieh and Wang, 2003; Blessing et al., 2012). Drawing from the cognitive intervention methods mentioned earlier, affective reminiscence involves recollecting personal memories that evoke positive emotions like joy, happiness, love, and gratitude. The objective is to enhance the mood, self-confidence, social interaction, and cognitive abilities such as memory, attention, language, and executive function of senior citizens (Goldwasser et al., 1987). Affective reminiscence can activate the hippocampus and other memory-related parts of the brain, thereby improving neurogenesis and synaptic plasticity (Cotelli et al., 2012). There has been a notable increase in research interest regarding the brain as a network. This trend is evident in the works of Varley and Sporns (2022) and Rutkowski et al. (2023a), as well as in the examination of brainwave time-series using a topological data analysis (TDA) by Varley et al. (2021). This innovative approach to TDA allows for the evaluation of various stages of conscious awareness in the brain, as demonstrated in studies involving anesthetized animals (Varley et al., 2021) and the prediction of age-related cognitive decline in humans (Rutkowski et al., 2023b).

Our research aims to explore the potential of using EEG biomarkers to predict MCI by testing working memory and evaluating conscious awareness in an experimental paradigm. As health neuroscience evolves, EEG-based neuro-biomarkers offer a reliable means of studying the impacts of reminiscent and affective stimulation. Our ongoing study can provide valuable insights into the prevention of dementia and the identification of relevant neural mechanisms. Our previous findings are documented in multiple publications (Rutkowski et al., 2021b, 2022a, 2023a,b). The current project uses innovative experimental paradigms to study affective reminiscent and working memory, distinct from the state-of-the-art resting state EEG (rsEEG) experiments (Babiloni et al., 2021). Additionally, the TDA feature extraction has been expanded beyond the original work by Varley et al. (2021), and has been applied to machine learning classification and regression models for MCI prediction. The main objective of the brief research report is to demonstrate the feasibility of using TDA features as potential neuro-biomarkers for MCI prediction rather than optimizing machine learning methods.

The brief research report paper is organized as follows: we introduce the methods developed within the presented project in the subsequent section, followed by the results presentation and the discussion summarizing the paper.

## 2 Method

In the summer of 2022, EEG experimental data was collected from older adult volunteers at Nicolaus Copernicus University in Torun, Poland. The Institute of Psychology UNC Ethical Committee for Experiments with Human Subjects endorsed the investigation. The experimental procedure and information collection followed The Declaration of Helsinki, which regulates ethical principles for research involving human subjects, including investigating identifiable human material and data. In a study conducted on 23 older adults, with an average age of 70.70 ± 5.32 years (refer to Supplementary Figure 1 for age distribution), 16 were diagnosed with Mild Cognitive Impairment (MCI). At the same time, the remaining 7 showed healthy cognitive aging. In the current post-pandemic pilot study, the cognitive abilities of all participants were evaluated using only the Montreal Cognitive Assessment (MoCA) paper and pencil test (Julayanont et al., 2012). A licensed evaluator administered the test. See Supplementary Figure 2 for a detailed distribution of MoCA scores. All participants willingly took part in the study and signed informed consent forms. The group mainly consisted of female participants, with only one male.

### 2.1 Reminiscent interior photography oddball paradigm

A reminiscence involves recalling past experiences and events, which can help define one's identity by connecting them with the future (Pinquart and Forstmeier, 2012; Woods et al., 2018; Buzsáki et al., 2022). Utilizing tangible audiovisual aids such as photos, music, or videos to communicate past life events is known as reminiscence intervention or stimulation (Thomas and Sezgin, 2021). Our study is based on the previous research conducted by our team on EEG brainwave patterns, as outlined in references (Rutkowski et al., 2021a, 2022b, 2023a). We plan to investigate how healthy aging or MCI affects the working memory of older adults using an interior photography oddball task. Additionally, we aim to establish a neuro-biomarker by measuring their conscious awareness during the task. In each quick trial, the participants view eight interior photographs encompassing contemporary and childhood-themed (reminiscent) scenes. Like the traditional oddball task, each image is designated as a target once, necessitating the participants to remember it before each trial. EEG data is consistently collected throughout the study, with triggers marking all phases for the 23 senior citizens who participated in the research. Every participant session comprises eight oddball sessions, each containing eight interior images arranged randomly, with four evocative of childhood and four featuring contemporary rooms.

**Figure 1:**
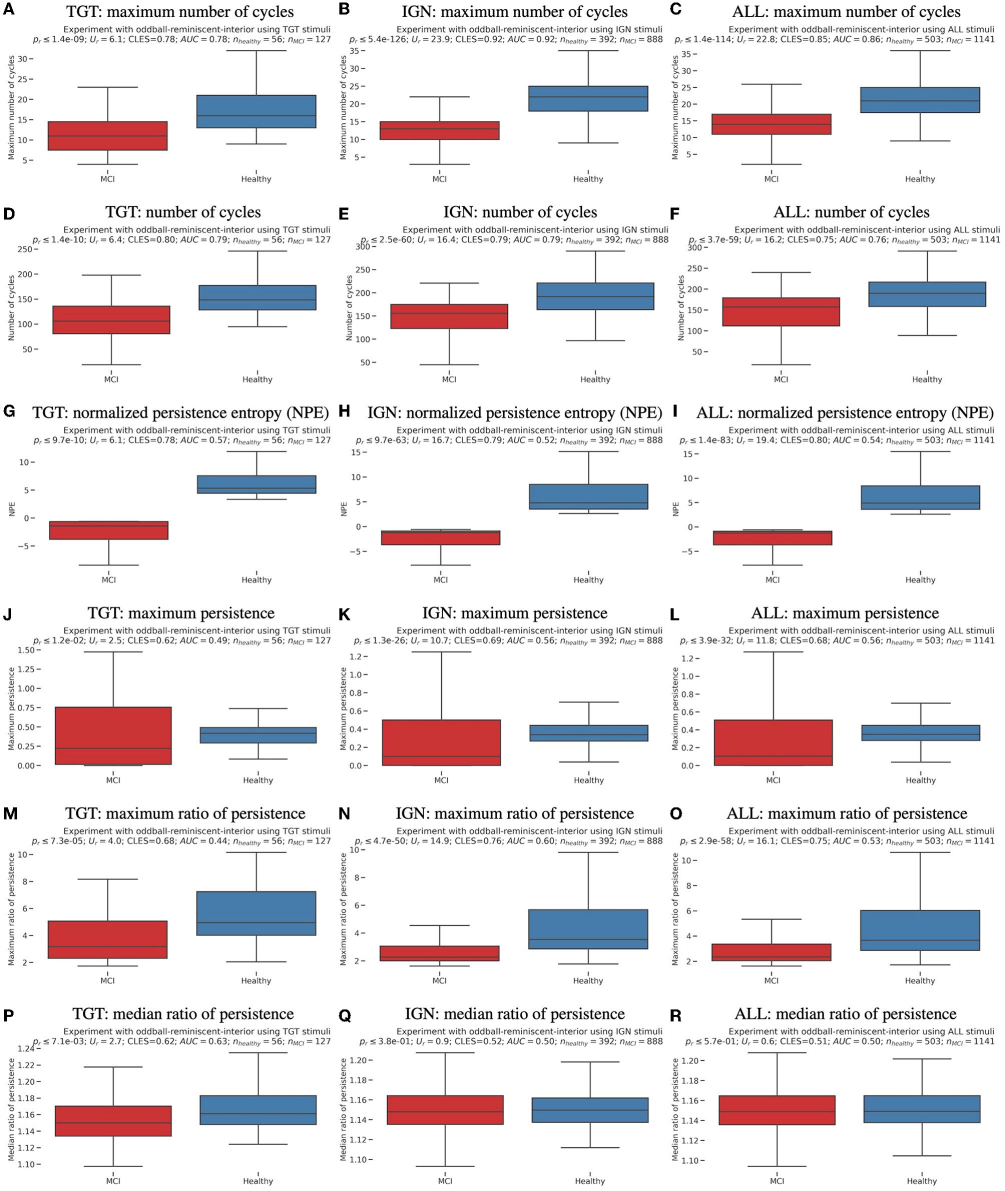
Boxplots with marked median, quartile ranges, and whiskers extending to show the rest of the distributions (majority non-normal distributions), together with Wilcoxon rank-sums test *p*−values, U-statistics, common language effect sizes (CLES) (McGraw and Wong, 1992) and area under the ROC curve (AUC) (Hanley and McNeil, 1982) scores for TDA features, used in subsequent unsupervised clustering, classification and regression, in three experimental response settings of the oddball paradigm's target (TGT), ignored (IGN), and all (ALL) stimuli arranged in columns. The sample numbers in both subject groups are marked by *n*_*healthy*_ and *n*_*MCI*_, respectively.

During the experimental session, there were a total of eight oddball sessions. Each oddball session included the presentation of one interior image (the target) followed by eight presentations of a target photograph randomly placed. This resulted in 72 responses from each participant. When excluding responses with missing markers due to stimulus system or network errors, there were 503 responses from healthy participants and 1141 responses from MCI participants. These values are denoted as *n*_*healthy*_ and *n*_*MCI*_ in Figures 1C, F, I, L, O, R in the all-stimulus-response case of Figure 1. Supplementary Section 1 provides a more detailed description of the experimental task procedure.

### 2.2 EEG experiments

Our study involved collecting EEG data using the Unicorn EEG headset from g.tec Medical Engineering, a reputable company based in Austria. Our prior research has demonstrated the reliability of this device compared to other available wearables, as reported in publications by Rutkowski et al. (2022c, 2023a). The preliminary investigation utilized eight EEG channels that uniformly cover the human scalp at standard locations such as Fz, C3, Cz, C4, Pz, PO7, Oz, and PO8. During the first preprocessing stage, we digitize the eight EEG streams at a sampling frequency of 250 Hz. Next, we apply a bandpass filter to eliminate signal baseline shifts and high-frequency noise within a frequency range of 1 to 40 Hz. Following this, we segment (or “epoch”) the EEG signals into 2-s time segments for reminiscent interior photography oddball tasks. These segments are determined using recorded triggers from the onset of each reminiscent photography stimulus. For our procedures in filtering and segmentation, we utilized the MNE package version 1.5.1 (Gramfort et al., 2013) in Python 3.11.5. To eliminate any artifacts caused by eye blinks or muscle movements in the EEGs we collected, we utilized a methodology previously developed by members of our research group (Rutkowski and Mori, 2015; Rutkowski et al., 2023a). The empirical mode decomposition (EMD) technique (Rutkowski et al., 2008, 2010) is utilized to purify EEG channels to break them down into intrinsic mode functions (IMF). Prior to reconstructing the final signal from sub-threshold IMFs, components exceeding 100 μV are eliminated (see Supplementary material). This entire process is carried out using PyEMD ver.1.5.1. Once the cleaning is complete, the resulting EEG traces are fed into the topological data analysis (TDA) application (Rutkowski et al., 2023b) for time series-based feature extraction. Please refer to the following section for a more comprehensive understanding of this process.

### 2.3 TDA processing of EEG

Topology in mathematics studies shapes and spaces, while topology in data analysis helps classify complex datasets by extracting topological invariants (Carlsson, 2009; Patania et al., 2017; Perea, 2018). Among the many topological methods developed for data analysis (Carlsson, 2009; Patania et al., 2017; Perea, 2018), a persistent homology is the most often used (Varley et al., 2021). Persistent homology permits the construction of descriptors of an embedded point cloud (EPC) shape and cataloging the existence of different structural features, such as connected components, cycles, and voids. In this study, we describe the noisy EPCs generated from an eight-channel EEG time series captured using a Unicorn EEG wearable with dry electrodes during an affective reminiscent interior photography oddball task performed by elderly participants. We utilize features such as persistent homology, number of cycles, and normalized persistence entropy, developed by Varley et al. (2021). This study builds upon our previous endeavor to classify MCI by utilizing network neuroscience features, as detailed in the publication by Rutkowski et al. (2023a). The ripser library version 0.6.4 (Tralie et al., 2018; Bauer, 2021) is used for TDA feature extraction, except for the newly introduced persistence ratios' analysis. The EPC obtained from eight EEG channel time series in each 2-s time segment is scrutinized to identify the maximum and total number of cycles, normalized persistence entropy (NPE), and maximum and median persistence ratios. Two novel improvements have been made to the original TDA methodology proposed by Varley et al. (2021): the use of latter ratio features and EMD-based EEG preprocessing. More details can be found in the Supplementary material. Figure 1 illustrates all these features.

### 2.4 Machine learning for MCI classification and MoCA regression

Our study focused on predicting MCI using TDA feature extraction and machine learning techniques. To visualize TDA features, we employed uniform manifold approximation and projection (UMAP) (McInnes et al., 2018), which allowed us to differentiate between cognitive classes without supervision. We did not need to preprocess with UMAP before applying two machine learning algorithms directly. We aimed to classify healthy cognitive aging and MCI and predict MoCA scores with limited participants. We utilized leave-one-out-subject cross-validation (LOOSCV) to achieve this, training a model using all participants except one. Our final results, including median and confidence intervals, were obtained by combining the results from each participant, as discussed in the Section 3.

To report binary classification results between healthy cognitive aging and MCI, we employed a random forest classifier (RFC) with a maximum of 200 trees in the forest. Further information about how the classifier performed in comparison to other methods is available in the Supplementary material. This classifier can be found in the scikit-learn ver. 1.3.0 library (Pedregosa et al., 2011). Additionally, we used a random forest regressor (RFR) to predict the exact MoCA score of each participant, using the same parameters as previously mentioned. Our machine-learning approaches relied on input features such as maximum and total number of cycles, normalized persistence entropy, maximum persistence, maximum ratio of persistence, and median.

## 3 Results

Our team's proposed affective reminiscence paradigm leverages TDA-drawn features from EEG and interior photography to reveal significant differences between healthy cognitive aging vs. MCI participants. Our visualization of these differences utilizes unsupervised clustering UMAP. The following sections comprehensively present our results from feeding the TDA features into random forest classifiers and regressors.

### 3.1 Topological data analysis feature distribution results

We utilized an oddball paradigm to evaluate working memory through TDA features extracted from EEG signals in three different contexts: targets (interior photographs that were instructed to be memorized in each trial), ignored photographs (also known as distractors), and all responses grouped. This proposed paradigm of affective reminiscence enabled us to conduct our analysis effectively. The results of the TDA-drawn features can be seen in Figure 1. The distributions were analyzed using non-parametric Wilcoxon rank-sum tests, which showed significant differences with a probability of *p*_*r*_≪0.01, except for the median ratio of persistence cases. It is worth noting that most of the distributions did not pass normality tests (with *p*_*n*_ < 0.05). As a result, we utilized reliable common-language-effect-size (CLES) (McGraw and Wong, 1992) and area under the ROC curve (AUC) (Hanley and McNeil, 1982) evaluations to support non-parametric statistical significance outcomes. These evaluations are indicated above each panel in Figure 1. According to our research, MCI individuals and anesthetized animals (Varley et al., 2021) displayed comparable outcomes concerning the number of filtration cycles and their maximum results (see Figures 1A–F). This suggests that people with MCI might have reduced levels of conscious awareness, as reflected in their EEG brainwave patterns. Moreover, while the study by Varley et al. (2021) we referred to did not yield any significant differences in maximum persistence, our findings revealed significantly higher outcomes for healthy cognitive aging participants, as illustrated in Figures 1J–O. Finally, the healthy cognitive aging group had significantly higher outcomes than the MCI group for a normalized persistence entropy (see Figures 1G–I). The encouraging differences in result distribution prompted us to explore unsupervised clustering and machine learning, with the outcomes discussed in the following sections.

### 3.2 Unsupervised clustering results

**Figure 2:**
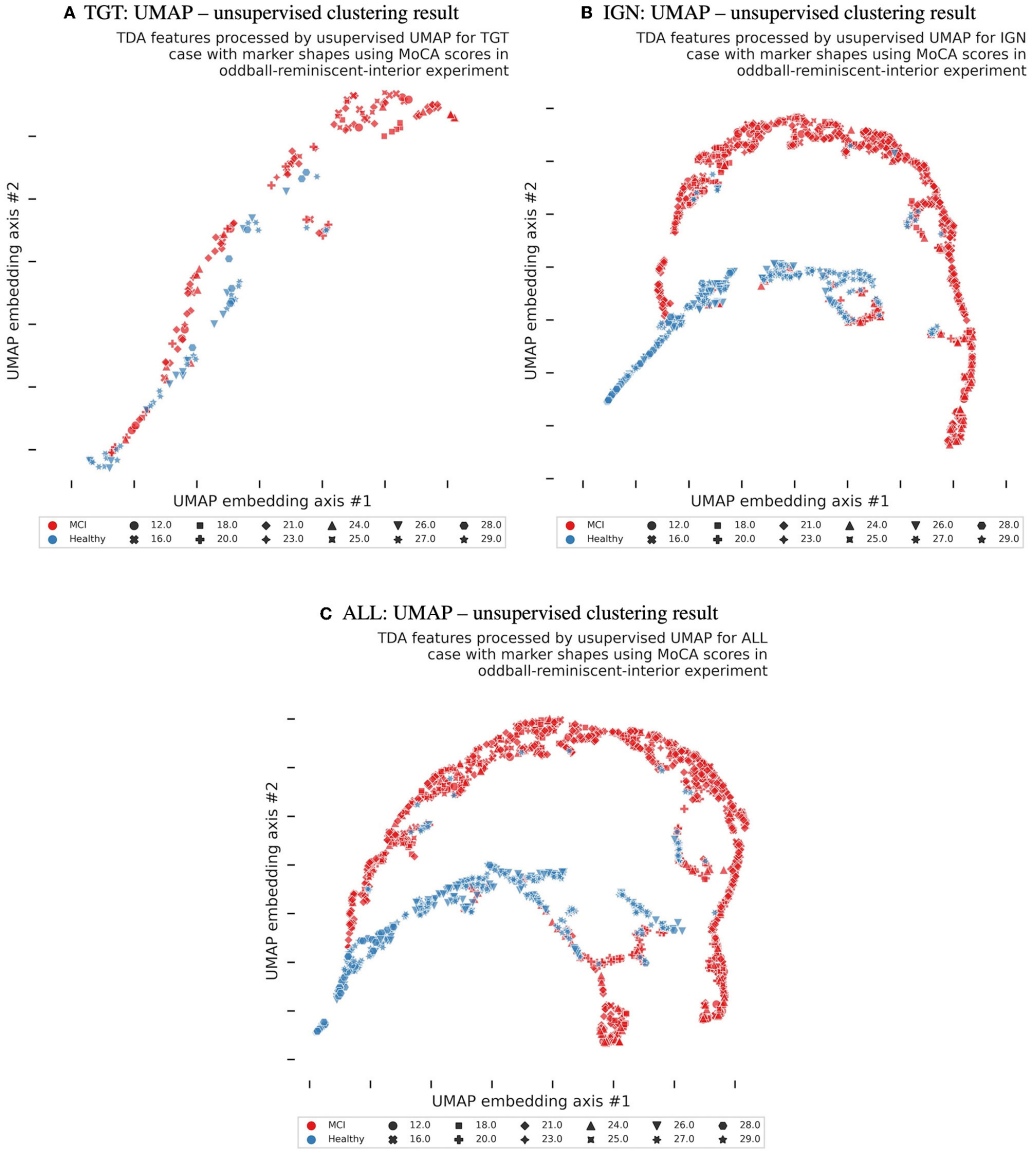
Unsupervised clustering (a machine learning training without class labels) scatter plots using UMAP (McInnes et al., 2018) in three experimental tasks and original data without any data augmentation.

Each of the three experimental results - targets (TGT), ignored (IGN), and all (ALL) brain responses grouped - were separated and analyzed using a clustering technique. The resulting findings are displayed in Figure 2, with color coding indicating MCI vs. healthy cognitive aging cases and marker shapes representing the exact MoCA scores. The UMAP unsupervised clustering projection on a two-dimensional plane made it easy to differentiate between cases of MCI and healthy cognitive aging. However, subsequent classification or regression would require non-linear methods. To compare and verify the results, we conducted a t-distributed stochastic neighbor embedding (t-SNE) (Van der Maaten and Hinton, 2008). This is presented in Supplementary Figure 5. Although both UMAP and t-SNE maintain the structure of data, UMAP is better at preserving the global structure, while t-SNE excels at preserving the local structure. Additionally, UMAP is more reliable and consistent than t-SNE, as it generates comparable results for the same data even with different random seeds and settings. This can be observed by comparing in Figures 2B, C and Supplementary Figure 5. Promising findings were yielded from examining 23 individuals using the affective reminiscent interior oddball paradigm. These findings substantiate the theory that TDA implementation holds great potential as a neuro-biomarker for MCI. Additionally, the examination exposed a connection to a reduction in conscious awareness in MCI subjects, a phenomenon noted in past animal anesthesia experiments (Varley et al., 2021). Further research is necessary to confirm preliminary findings. Ideally, a multicultural group should be studied focusing on MCI level.

### 3.3 MCI prediction results

**Figure 3:**
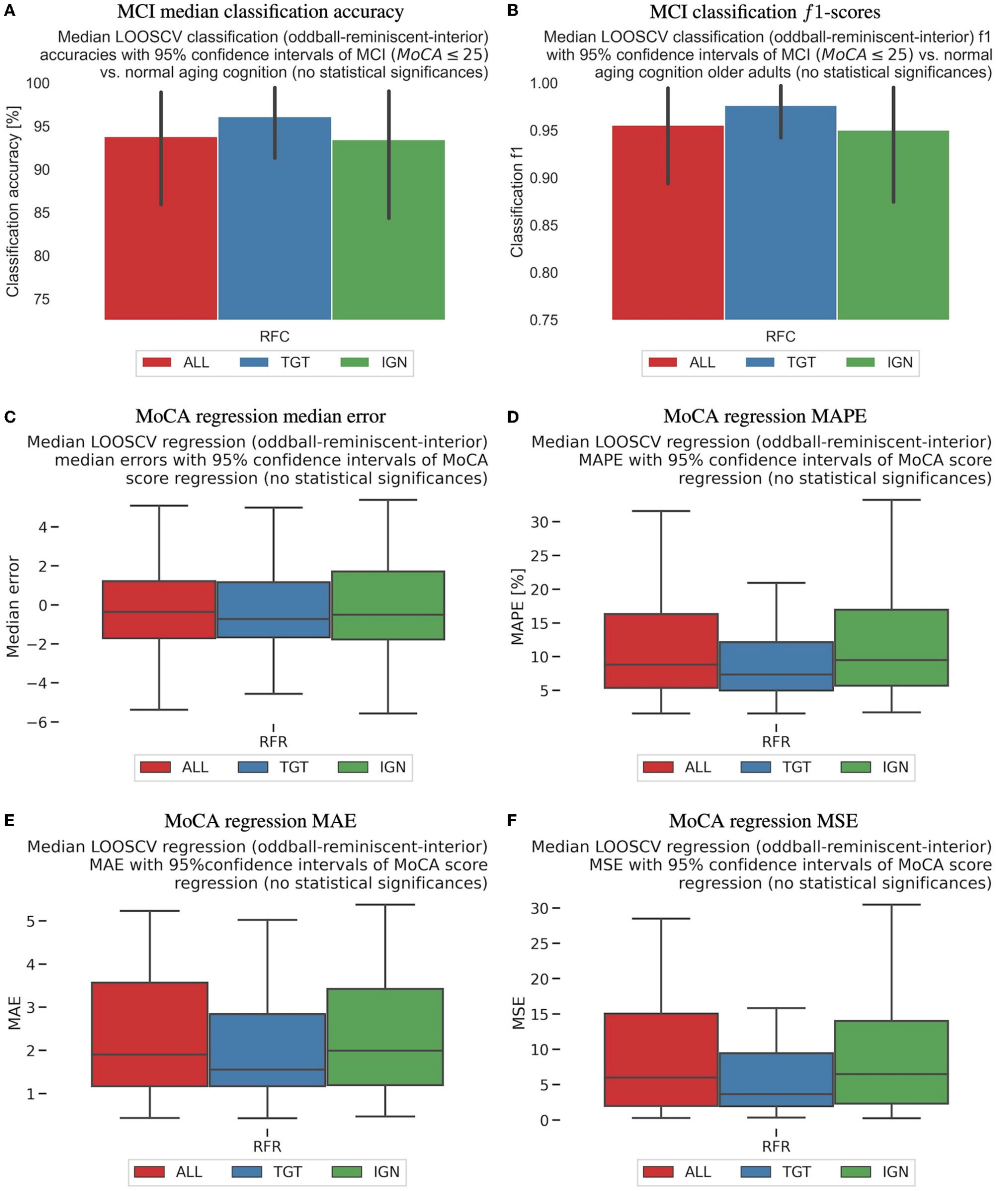
The boxplot diagrams show the median, quartile, and 95−percentile ranges of LOOSCV-based MCI classification in **(A, B)**, as well as MoCA regression results in **(C–F)**. The chance level is set at 70% for classification results due to unequal class memberships (MCI vs. healthy). Accuracy is displayed in **(A)**, while **(B)** shows *f*1−scores. Regression median errors for exact MoCA-score prediction are presented in **(C)**, mean absolute percentage errors (MAPE) in **(D)**, mean absolute errors (MAE) in **(E)**, and mean squared errors (MSE) in **(F)**.

The first section of Table 1 and the upper panels of Figure 3 provide a comprehensive overview of the successful outcomes of the classification tests conducted on healthy cognitive aging vs. MCI across three response settings: targets, ignored, and all. The median accuracies for all response groups exceeded 93%, with no noteworthy disparity between them. The chance level for classification accuracy was set at 70% due to unequal class memberships between MCI and healthy cognitive aging groups. Furthermore, the classification results were corroborated by impressive AUC, *f*1, precision, and recall scores, as detailed in Table 1.

**Table 1:** LOOSCV overall MCI prediction and MoCA-score regression results.

**LOOSCV overall MCI prediction (a binary classification of MCI vs. normal) results**					
**Stimulus case**	**Accuracy**	**AUC**	*f*1	**Recall**	**Precision**
Target (TGT)	95.63%	0.96	0.96	0.96	0.96
Ignored (IGN)	93.13%	0.90	0.93	0.93	0.93
All (ALL)	93.86%	0.92	0.94	0.94	0.94
**LOOSCV overall MoCA-score regression (the exact MoCA score prediction) results**					
**Stimulus case**	**r2**	**MSE**	**MAE**	**Median error**	**MAPE (%)**
Target (TGT)	0.35	11.53	2.52	0.37	13.21
Ignored (IGN)	0.32	12.14	2.66	0.21	13.68
All (ALL)	0.34	11.80	2.59	0.34	13.37

### 3.4 MoCA regression results

We attempted a regression analysis after successfully classifying data in the binary setting as reported in Section 3.3. However, these findings should be interpreted cautiously due to a limited number of participants and unequal distribution of MoCA scores, as shown in Supplementary Figure 2. The results are shown in Table 1 and Figures 3C–F. The regression *r*2 score of the model exceeded that of a naive regressor, indicating its ability to account for over 30% of the variability in the dependent variables. Encouragingly, the mean absolute error (MAE) produced even more impressive findings, accurately forecasting the subject's MoCA score within a narrow range of ±2.6. Moreover, in the LOOSCV setting, the subjects' median errors were below 0.4, indicating that the TDA analysis performed in the affective reminiscence paradigm holds the promise of being a reliable neuro-biomarker candidate with a broader participant pool. Furthermore, all cases exhibited mean absolute percentage error outcomes less than 14%, reinforcing our research approach's soundness. According to Figures 3C–F, there were no significant differences in the regression results between target, ignored, and all reminiscent photography stimuli.

## 4 Discussion

Previously, Varley et al. (2021) have utilized network neuroscience analysis to investigate brainwave time series and their correlation with levels of conscious awareness. Varley et al. (2021) research established a relationship between the network features and the degree of conscious awareness in animals undergoing anesthesia. Similar observations have been shown in EEG by Rutkowski et al. (2023a), also using a similar network neuroscience approach for forecasting MCI in human subjects; however, the relation to awareness modulation has yet to be fully established. The same previous study by Varley et al. (2021) used TDA analysis of brainwave time series to identify statistical differences in the number of filtration cycles and their maximum results in anesthetized animals. Our current study found that participants with MCI have a statistically significant decrease in the number of cycles during filtration, indicating similarity to the lower awareness levels of anesthetized animals (Varley et al., 2021). This preliminary finding, based on a limited subject group, suggests a potential link between MCI and lower conscious awareness levels in elderly individuals. However, further experimental confirmation beyond the oddball paradigm is necessary to confirm this hypothesis.

According to the results of the current study, healthy cognitive aging participants have higher conscious awareness levels during the experimental task. This is reflected in the significantly higher numbers of cycles and maximum persistence ratios observed in the EEG of these participants. This finding is consistent with a previous study by Varley et al. (2021), which showed similar results with pharmacologically controlled anesthesia levels. These results suggest that healthy aging brains exhibit more frequent state transitions (Barabási et al., 2023) during cognitive tasks that involve affective reminiscence and working memory. This may indicate that older individuals exhibit greater awareness during tasks, as demonstrated by significantly higher numbers of cycles and maximum persistence ratios observed in EEG, related to more reach state transitions (Sizemore et al., 2019).

As highlighted in Section 3.2, the dissimilarities in distributions that were detected via TDA analysis were validated to have statistical significance with the aid of unsupervised machine learning clustering techniques like UMAP and t-SNE. The obtained clusters exhibited non-linear separability, indicating their potential usefulness in non-linear classification and regression approaches that may follow.

The final LOOSCV classification experiment presented in Section 3.3 resulted in solid and above 93% classification accuracies supported significant *f*1-scores, as well as safely above the 70% chance level in the study. Three different response groups of selected only targets or ignored reminiscent interior photographs, as well as all the above grouped, resulted in non-significant classification and regression results as discussed in Sections 3.3, 3.4.

The presented brief research report has a limitation regarding participant numbers, with only 23 individuals included. Additionally, the class membership is unbalanced, with a significantly higher number of individuals with Mild Cognitive Impairment (MCI), as evaluated only with MoCA tests, than those with healthy cognitive aging. Moreover, the study had limited gender diversity, with only one male participant. As we move forward, we plan to conduct a project involving a larger group of participants with a balanced gender distribution. This project may be undertaken in cross-cultural settings to validate and reproduce the findings. Additionally, we intend to include more cognitive tests to support our findings, as stated by Hodges and Larner (2017). An effective means of enhancing the present research lies in solidifying the linkages between the anticipated phases of MCI and the cognitive scores (Hodges and Larner, 2017; Nara et al., 2018). Accomplishing this requires the integration of supplementary assessments, such as PET and cerebrospinal fluid (CSF) biomarkers for Alzheimer's syndrome or structural MRI for evaluating vascular dementia (Morinaga et al., 2010; Bucci et al., 2021). By doing so, the suggested neuro-biomarker will become more dependable. Our study delves into applying topological data analysis (TDA) techniques in scrutinizing multivariate EEG time series. Our primary focus is to uncover the disparities between age-related healthy cognitive function and MCI, as well as the regression of cognitive scores, with the aid of TDA. We accomplish this by analyzing the temporal dynamics of all EEG channels and constructing multi-dimensional shapes using embedded point cloud (EPC) data. Our findings reveal how the brain progresses through state space over time (Sizemore et al., 2019; Barabási et al., 2023; Betzel et al., 2023). The study aims to develop an inexpensive neuro-biomarker for monitoring cognitive interventions and subsequent dementia care remotely.

## Data availability statement

The datasets presented in this article are not readily available because the subject consent form did not include permission to share the EEG data outside the current project. Requests to access the datasets should be directed to tomasz.rutkowski@riken.jp.

## Ethics statement

The studies involving humans were approved by the Institute of Psychology UNC Ethical Committee for Experiments with Human Subjects. The studies were conducted in accordance with the local legislation and institutional requirements. The participants provided their written informed consent to participate in this study.

## Author contributions

TR: Conceptualization, Data curation, Formal analysis, Funding acquisition, Investigation, Methodology, Project administration, Resources, Software, Supervision, Validation, Visualization, Writing - original draft, Writing - review & editing. TK: Data curation, Investigation, Methodology, Validation, Writing - review & editing. MO-M: Funding acquisition, Project administration, Resources, Supervision, Writing - review & editing.
