# Supplementary material for: Mild cognitive impairment prediction and cognitive score regression in the elderly using EEG topological data analysis and machine learning with awareness assessed in affective reminiscent paradigm
Source: Front Aging Neurosci. 2024 Jan 4;15:1294139. doi: 10.3389/fnagi.2023.1294139 (PMC10794306; doi:10.3389/fnagi.2023.1294139)
Supplement: Supplementary file 1 [file Data_Sheet_1.PDF]

Supplementary Material

1 EXPERIMENTAL PARTICIPANTS' STATISTICS

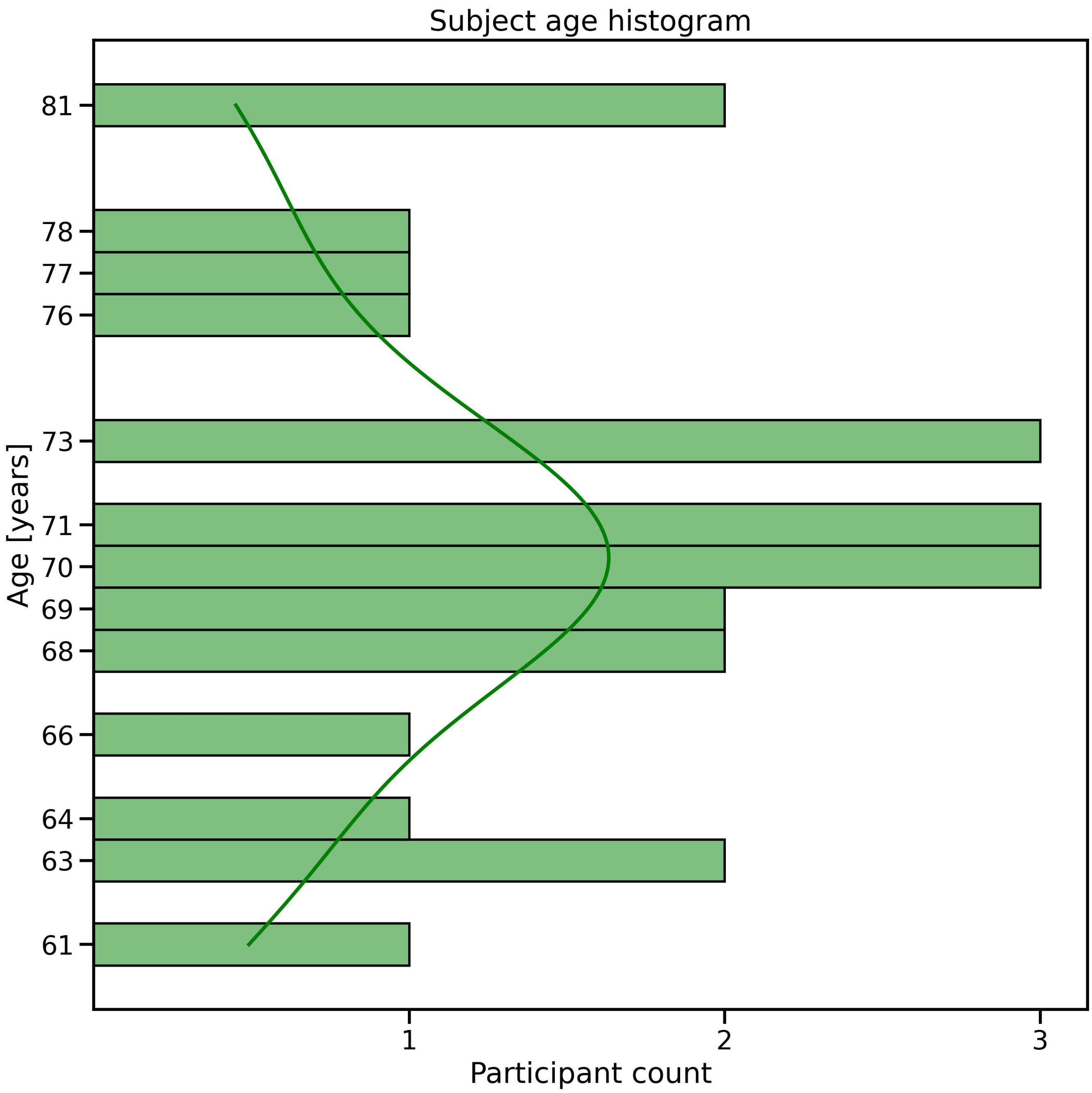

Figure S1. The elderly 23 participants' age histogram. The mean age of 70.70 ± 5.32 years old.

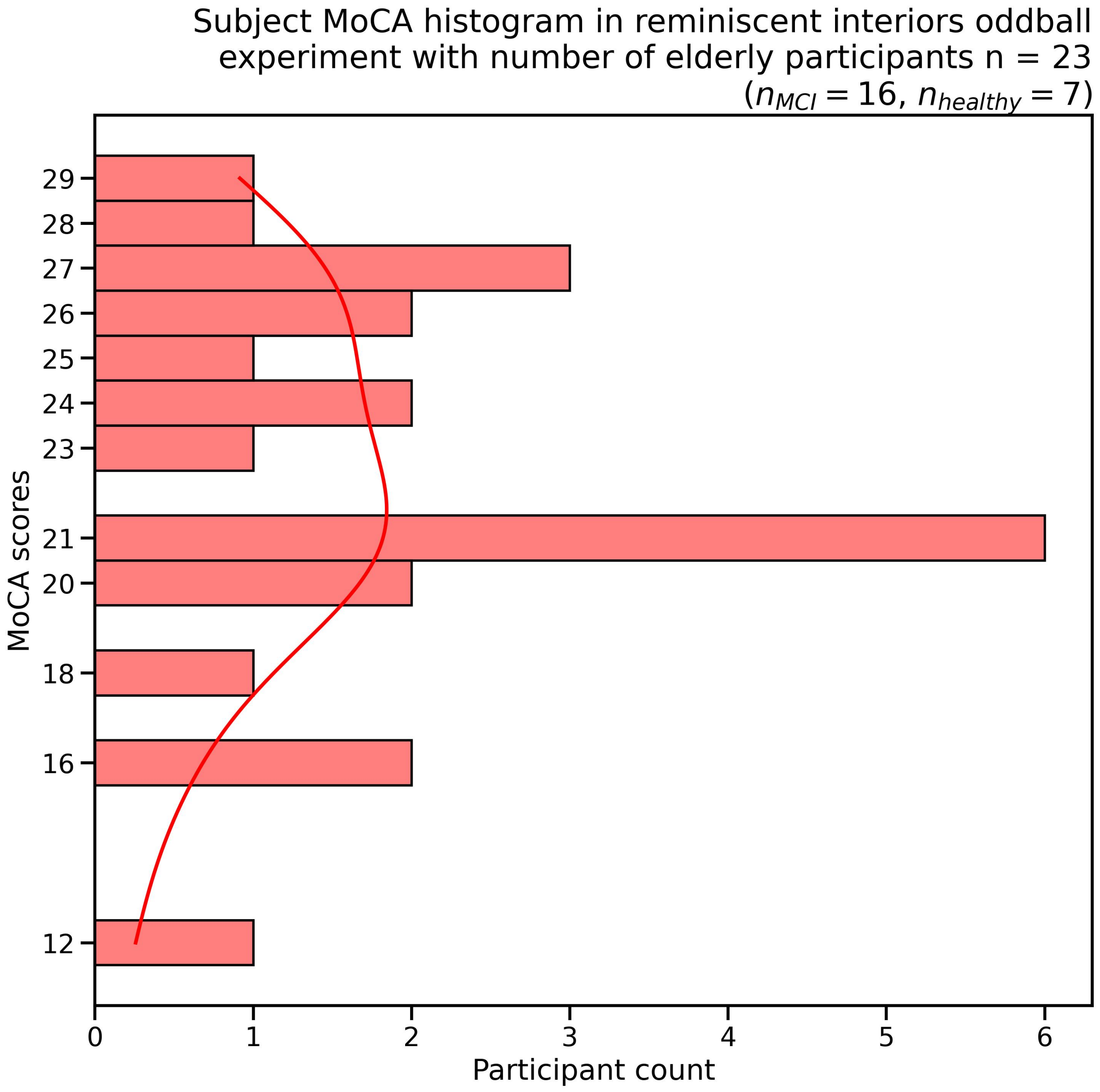

Figure S2. The elderly 23 participants' histogram of MoCA scores in the reminiscent room design picture oddball task. Mean MoCA score of 22.35 ± 4.22.

2 DETAILS OF THE EXPERIMENTAL TASK

Our research builds on prior studies conducted by our team on EEG brainwave patterns, which are outlined in references (Rutkowski et al., 2021, 2022, 2023).

Prior to EEG recording sessions, we obtained informed consent from each elderly participant, explaining the experimental procedure and its purpose. We displayed eight types of high-quality, colorful interior pictures during each recording session on a 23-inch computer display. The images were presented in Japanese and Polish styles and categorized as either old (post World War II) or modern (recent designs), with two examples from each category.

We used an oddball-style paradigm (Donchin and Coles, 1988; Wolpaw and Wolpaw, 2012) where each participant was instructed to memorize one image presented for two seconds before each eight-image random sequence presentation. After a single-second break, we introduced a random sequence of seven distractors (non-targets to be ignored) with a single memorized (target) image. The presentation of each image lasted two seconds, followed by a single-second break (black screen with a white fixation cross in the center). Thus, the stimulus onset asynchrony (SOA) was fixed at three seconds in each eight-image presentation trial, followed by another single-second break and two seconds of instruction for the next trial target. Each interior photograph from the series of eight became a target once, and a single experimental session consisted of 64 stimulus presentations with a target to remember, adjusting each time after eight random appearances.

Before each EEG recording session, we conducted a brief explanation and behavioral trial for the elderly participants to help them become familiar with the procedure and the stimulation environment. During the training, we displayed a sequence of four images, with each image serving as an instructed target. We asked each participant to press a button to confirm their understanding of the procedure and indicate that they had successfully encoded the target image in memory. In the explanation trials, we used MAX (Cycling ’74, 2019), a visual programming environment, to present stimuli and collect behavioral responses via a touchscreen tablet. All participants completed the training sessions. During the EEG recording trials, the participants were only required to mentally confirm the targets without pressing any buttons.

3 DETAILS OF EEG PREPROCESSING

3.1 EEG Bandpass Filtration

Our in-house EEG recording system generated triggers for post-processing and to identify onsets and offsets of EEG time segments. These segments were then pre-processed in Python 3.11.5 using version 1.5.1 of the MNE package. We applied a band-pass filter with a range of $1 \sim 40$ Hz to filter out any unwanted noise using IIR forward-backward fourth-order Butterworth filters. Each EEG channel undergoes independent filtering stages, as shown in Figure S3.

3.2 EEG Single-channel EMD-based Artifact Filtration

To extract detailed information from single experimental trial signals without removing muscular artifacts from EEG signals, we use empirical mode decomposition (EMD) (Laszuk, 2017). This approach has been previously explored in literature by Rutkowski et al. (2010); Rutkowski and Mori (2015).

The EMD approach involves identifying nonstationary and nonlinear brain activity features captured by EEG data acquisition system (Rutkowski et al., 2008a,c,b). These features are extracted as intrinsic mode functions (IMFs), explained in this section’s latter part. For this application, we propose to examine

the level of detail revealed in single EEG channels that are decomposed separately into IMFs. We then apply a threshold to these IMFs using absolute values of maxima exceeding a value of 100 μV, traditionally recognized as a non-EEG source related to EMG or EOG muscular activities (Schomer and Lopes da Silva, 2011). According to a study by Huang et al. (1998), all the IMFs create semi-orthogonal bases from the original EEG signals and are not introduced artificially by the method itself. The IMF components obtained during EMD analysis should meet the requirements of completeness, orthogonality, locality, and adaptiveness. Getting an IMF involves removing local riding waves and asymmetries estimated from the waveform's local envelope of minima and maxima. There are several approaches to assessing signal envelopes (Huang et al., 1998). The finding IMFs involves finding limited-band signals and eliminating riding waves from the EEG. This ensures that the instantaneous frequency will not have fluctuations caused by an asymmetric waveform. Zero crossings define an IMF in each cycle. Every IMF involves only one oscillation mode; no complex riding waves are allowed. It is important to note that the IMF is not limited to being a narrow band signal as in traditional Fourier or wavelets decomposition. Instead, it can be both amplitude and frequency modulated simultaneously and nonstationary or nonlinear. To extract the intrinsic mode functions (IMFs) from a signal $x(t)$, a sifting process is used which involves the following steps: (1) identifying the local maxima and minima of the signal being analyzed; (2) generating the upper and lower signal envelopes by connecting those local maxima and minima, respectively, using a chosen interpolation method such as linear, spline, cubic spline, or piece-wise spline; (3) calculating the local mean $m(t)$ by averaging the upper and lower signal envelopes; (4) subtracting the local mean from the data using the following equation:

$$h_1(t) = x(t) - m_1(t). \tag{S1}$$

Ideally, $h_1(t)$ should be an IMF candidate, as in equation (S1). However, in practice, $h_1(t)$ may still contain local asymmetric fluctuations such as undershoots and overshoots. Therefore, one needs to repeat the four steps mentioned earlier to obtain the first optimized IMF. The sifting process is applied to the residue $\epsilon_1(t)$ to get the second IMF. This residue is obtained by subtracting the first IMF from $x(t)$. Similarly, the third IMF is extracted from the residue ϵ_2, and so on. The extraction of IMFs continues until two successive sifting results are nearly identical. The empirical mode decomposition (EMD) of a signal $x(t)$ can be represented as a sum of n intrinsic mode functions (IMFs) and a final residue $\epsilon_n(t)$, which can be either a constant or the mean trend. The equation can be written as follows:

$$x(t) = \sum_{k=1}^{n} IMF_k(t) + \epsilon_n(t), \tag{S2}$$

where n denotes the total number of extracted IMFs. The equation (S2) shows that the EMD is complete, resulting in an equality. The original signal can be reconstructed by adding all IMFs and the final residue. Although the IMFs are not guaranteed to be mutually orthogonal, they are often close to orthogonal in practice. Additionally, it is essential to note that IMFs are adaptive, meaning they depend on the signal $x(t)$. This is expected for a data-driven method.

To remove all non-EEG originating muscular artifacts, we use a thresholding approach to reconstruct the EEG only from IMFs that do not exceed a fixed threshold of 100μV, which means that any IMFs exceeding this threshold are removed (zeroed) from the reconstruction equation (S2) in each EEG channel separately. This simplified procedure, similar to our previous approaches (Rutkowski et al., 2008a,c,b), also successfully eliminates all muscular artifacts from noisy EEG. Figure S3 illustrates the stages of EMD-based muscular artifact rejection conducted independently on each EEG channel.

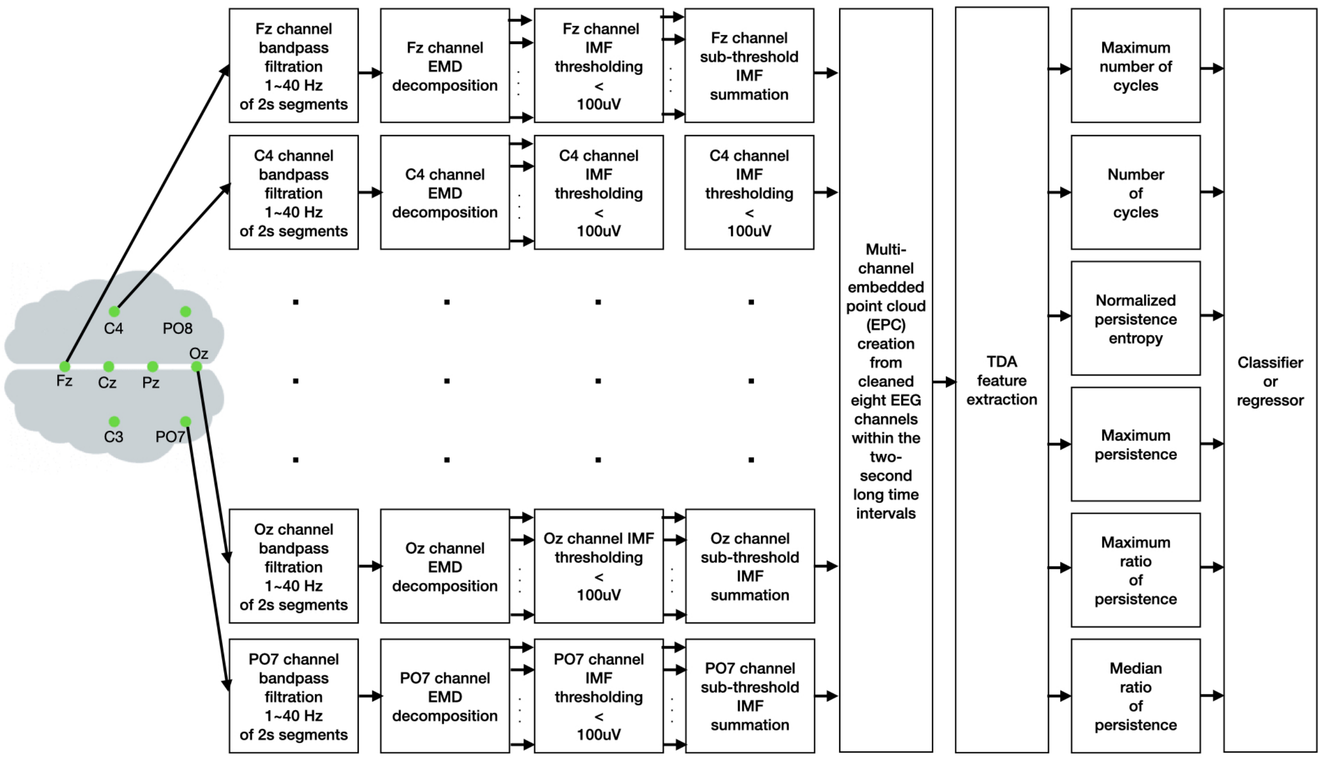

Figure S3. The EEG preprocessing and TDA feature extraction pipeline.

4 DETAILS OF TOPOLOGICAL DATA ANALYSIS APPLICATION TO MULTIVARIATE EEG FEATURE EXTRACTION

Below, we will discuss how topological data analysis (TDA) has been applied to extract multivariate EEG features. The focus will be on providing details about the process and the benefits of using this approach.

4.1 Embedded Point Cloud (EPC) from EEG Signals

In the EEG analysis, we studied the emergence and evolution of global activity patterns, represented as states in a high-dimensional space. This approach provided valuable insights into the system's underlying structure and allowed for a more precise quantification of its behavior. Each EEG recording comprises two-second-long time segments, which can be represented as a matrix $\mathbf{E}$ with dimensions $c \times s$. In our case, c is the number of EEG channels (eight channels), and s is the number of samples taken during the recording (500 samples). Each column-vector in $\mathbf{E}$ represents a distribution of micro-voltages across the recording array at each moment in time. In simpler terms, each column vector provides a snapshot of the electrical activity in the brain at a given moment during the EEG recording. To create the embedded point cloud (EPC), we consider every column as a vector in a space with c dimensions. This way, for each time stamp, the brain state can be represented by a c-dimensional vector within the space of all potential states it can have. As time elapses, the recording can be viewed as a trajectory in a continuous, c-dimensional state space. Each EPC is then analyzed for feature extraction using the TDA style, as described in the following section. The EPC block is depicted in Figure S3 to illustrate the processing stage in our EEG preprocessing pipeline.

4.2 Topological Data Analysis (TDA) Feature Extraction

Persistent homology is one of the most commonly used topological methods in TDA field (Varley et al., 2021). It enables us to create descriptions of the shape of a point cloud by recording the presence of various structural features, such as connected components, cycles, voids, and more (Tralie et al., 2018; Bauer, 2021). This is achieved by examining the existence of these features at various levels of coarse discretization of the data. The Rips filtration or Vietoris-Rips filtration process involves growing spheres centered on each embedded point, which intersect and connect the data points to reveal the shape of the data (Tralie et al., 2018; Bauer, 2021). As the sphere radius increases, data representation changes. Persistent homology measures changes by considering the number of connected components, when they merge, and when they form more complex structures. To compare features, we utilize Betti curves (Tralie et al., 2018; Bauer, 2021). A Betti curve is used for each feature type, such as connected components and cycles. It counts the number of features present at each scale in the Rips filtration (Tralie et al., 2018; Bauer, 2021). The curves are comparable because they all have the same support: the increasing radius of the spheres in the embedding space. These curves can be compared to one another or averaged together to determine the radius that generates the most complex feature sets. We consider the following TDA features, which serve as inputs to machine learning models and describe EEG time segments:

- **Maximum number of cycles:** In topological data analysis, the maximum number of cycles characterizes the complexity of a dataset. It is obtained by tracking the birth and death of topological features as the filtration parameter changes. The maximum number of cycles defines the most prominent feature in the dataset. It focuses on the overall complexity and richness of the topological structure in the data, counting the maximum number of features (cycles) at a particular scale.
- **Number of cycles:** A cycle count is obtained by tracking all births and deaths of topological features as filtration parameters change in topological data analysis.
- **Normalized persistence entropy (NPE):** Entropy measures disorder or uncertainty. In persistent homology, its diagram quantifies complexity in topological features across scales. Normalized entropy (NPE) is easily comparable across data sets and scales by dividing by the maximum value in the dataset.
- **Maximum persistence:** The most extended persistence interval observed in a persistence diagram is a crucial indicator of the most stable and significant topological features in the given data. Considering the most extended feature lifespan in the persistence diagram, it highlights the stability and robustness of individual topological features.
- **Maximum ratio of persistence:** A novel feature introduced in our approach compared to (Varley et al., 2021). Instead of a traditional interval used in maximum persistence interval, it is a ratio obtained by dividing the end (death) by the beginning (birth) of the longest interval. It results in a more robust measure used to compare the significance of topological features between different datasets or scales within the same dataset. Its values are considered more important or salient.
- **Median ratio of persistence:** A second novel feature introduced in our approach compared to analysis introduced by Varley et al. (2021). It is the median value of ratios obtained from analyzed EEG time segments, just like the maximum value described above.

The TDA features are shown in Figure S3, just before the final classification and regression stages discussed in the following section.

5 DETAILS OF MACHINE LEARNING APPLICATION

5.1 Classifier Settings

After conducting a short pilot study to compare the state-of-the-art simple classifiers available in the scikit-learn ver. 1.3.0 library (Pedregosa et al., 2011), and running calculations on a typical laptop computer, we decided to use a random forest classifier (RFC) due to the best results as compared in Figure S4. This brief research report aims to demonstrate the feasibility of TDA features as potential biomarkers for dementia rather than to optimize machine learning methods. We, therefore, used classical off-the-shelf methods from scikit-learn (Pedregosa et al., 2011). Below are details of each classification method with results summarized in Figure S4. We applied a standard scaler to standardize input features by removing the mean and scaling to unit variance for all tested methods, as also available in scikit-learn (Pedregosa et al., 2011). All classifier evaluation runs utilized leave-one-subject-out cross-validation (LOOSCV) with a chance level of 70%.

- **Logistic regression (LR) classifier** with the following default parameters: $penalty = l2$, $dual = False$, $tol = 0.0001$, $C = 1.0$, $fit\_intercept = True$, $intercept\_scaling = 1$, $class\_weight = None$, $random\_state = None$, $solver = lbfgs$, $max\_iter = 100$, $multi\_class = auto$, $verbose = 0$, $warm\_start = False$, $n\_jobs = None$, $l1\_ratio = None$.
- **Linear discriminant analysis (LDA) classifier** with the following default parameters: $solver = svd$, $shrinkage = False$, $priors = None$, $n\_components = None$, $store\_covariance = False$, $tol = 0.0001$, $covariance\_estimator = None$.
- **shrinkage LDA (sLDA) classifier** with the following default parameters $solver = lsqr$, $shrinkage = True$, $priors = None$, $n\_components = None$, $store\_covariance = False$, $tol = 0.0001$, $covariance\_estimator = None$.
- **Linear support vector machine (linearSVM) classifier** with the following default parameters: $C = 1.0$, $kernel = linear$, $degree = 3$, $gamma = 2$, $coef0 = 0.0$, $shrinking = True$, $probability = False$, $tol = 0.001$, $cache_size = 200$, $class\_weight = None$, $verbose = False$, $max\_iter = -1$, $decision\_function\_shape = ovr$, $break\_ties = False$, $random\_state = 42$.
- **Radial basis function SVM (RBF SVM) classifier** with the following default parameters: $C = 1.0$, $kernel = rbf$, $degree = 3$, $gamma = 2$, $coef0 = 0.0$, $shrinking = True$, $probability = False$, $tol = 0.001$, $cache_size = 200$, $class\_weight = None$, $verbose = False$, $max\_iter = -1$, $decision\_function\_shape = ovr$, $break\_ties = False$, $random\_state = 42$.
- **Random forest classifier (RFC)** with the following default parameters: $n\_estimators = 200$, $criterion = gin$, $max\_depth = None$, $min\_samples_split = 2$, $min_samples\_leaf = 1$, $min\_weight\_fraction\_leaf = 0.0$, $max\_features = sqrt$, $max\_leaf\_nodes = None$, $min\_impurity\_decrease = 0.0$, $bootstrap = True$, $oob\_score = False$, $n\_jobs = None$, $random\_state = 42$, $verbose = 0$, $warm\_start = False$, $class\_weight = None$, $ccp\_alpha = 0.0$, $max\_samples = None$.
- **Fully-connected neural network (FNN) classifier** with the following default parameters: $hidden\_layer\_sizes = (100,)$,$activation = relu$, $solver = adam$, $alpha = 0.0001$, $batc_size = auto$, $learning\_rate = constant$, $learning\_rate\_init = 0.001$, $power\_t = 0.5$, $max\_iter = 200$, $shuffle = True$, $random\_state = 42$, $tol = 0.0001$, $verbose = False$, $warm\_start = False$, $momentum = 0.9$, $nesterovs\_momentum = True$, $early\_stopping = False$,

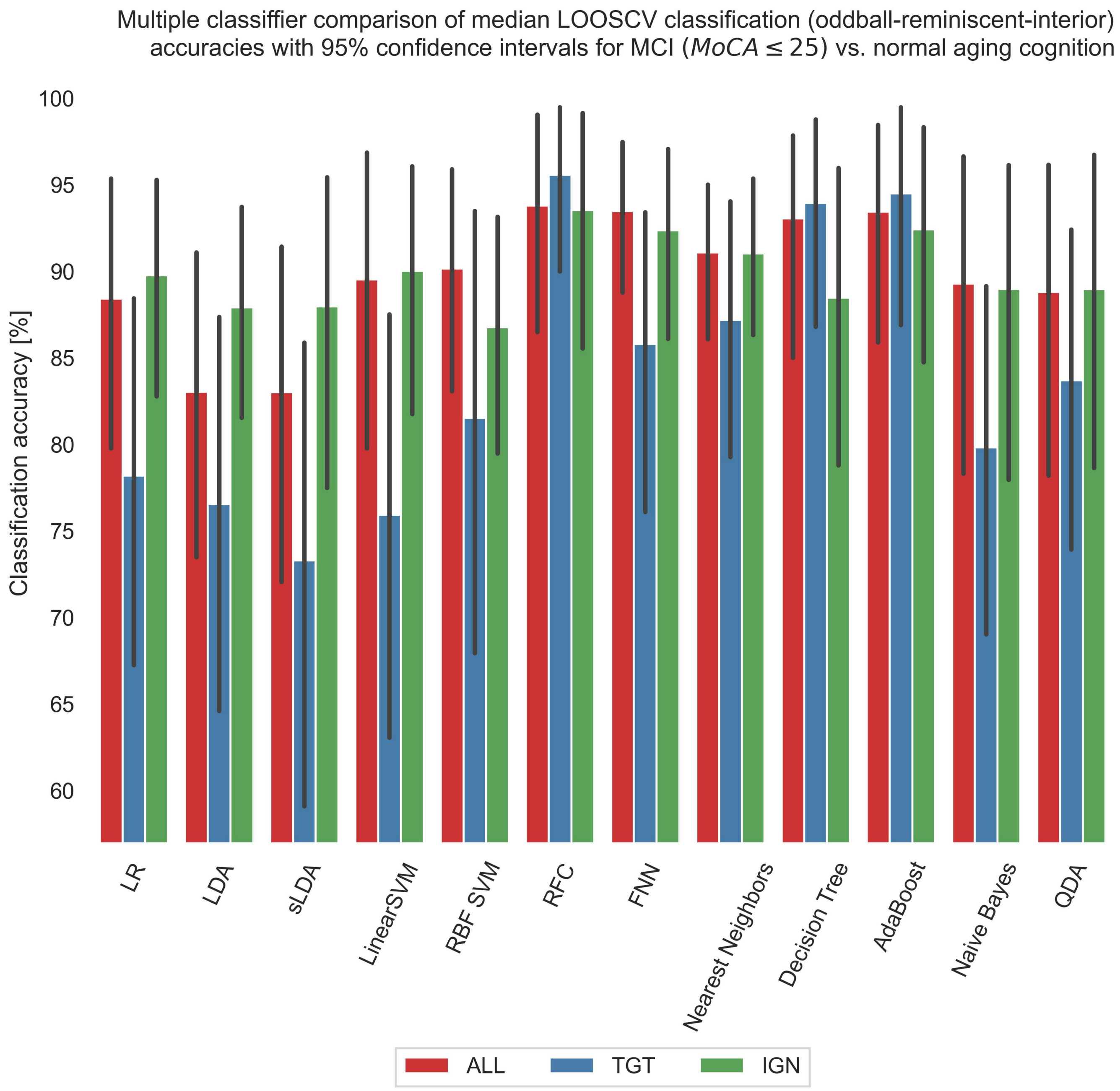

Figure S4. This study compared the performance of various machine learning algorithms in differentiating between individuals with mild cognitive impairment (MCI) and healthy aging cognition subjects. The accuracy of these algorithms was measured through leave-one-subject-out cross-validation (LOOSCV), with a chance level of 70%. The algorithms used in this study include logistic regression (LR), linear discriminant analysis (LDA), shrinkage LDA, linear support vector machine (linearSVM), radial basis function SVM (RBF SVM), random forest classifier (RFC), fully-connected neural network (FNN), nearest neighbors classifier, decision tree, AdaBoost, naive Bayes, and quadratic discriminant analysis (QDA). The performance of various algorithms was tested in their ability to distinguish between individuals with Mild Cognitive Impairment (MCI) and those with healthy cognitive aging. This was done by analyzing their response to affective reminiscent oddball targets (TGT), non-targets/ignored stimuli (IGN), and all stimuli (ALL). The results are presented in the form of comparison bar plots that display the mean accuracies along with 95% confidence intervals.

$validation\_fraction = 0.1$, $beta\_1 = 0.9$, $beta\_2 = 0.999$, $epsilon = 1e-08$, $n\_iter\_no\_change = 10$, $max\_fun = 15000$.

- **Nearest neighbors classifier** with the following default parameters: $n\_neighbors = 2$, $weights = uniform$, $algorithm = auto$, $leaf\_size = 30$, $p = 2$, $metric = minkowski$, $metric\_params = None$, $n\_jobs = None$.
- **Decision tree classifier** with the following default parameters: $criterion = gini$, $splitter = best$, $max\_depth = None$, $min\_samples\_split = 2$, $min\_samples\_leaf = 1$, $min\_weight\_fraction\_leaf = 0.0$, $max\_features = None$, $random\_state = 42$, $max\_leaf\_nodes = None$, $min\_impurity\_decrease = 0.0$, $class\_weight = None$, $ccp\_alpha = 0.0$.
- **AdaBoost classifier** with the following default parameters: $estimator = None$, $n\_estimators = 50$, $learning\_rate = 1.0$, $algorithm = SAMME.R$, $random\_state = 42$.
- **Gaussian naive Bayes (Naive Bayes) classifier** with the following default parameters: $priors = None$, $var\_smoothing = 1e-09$.
- **Quadratic discriminant analysis (QDA) classifier** with the following default parameters: $priors = None$, $reg\_param = 0.0$, $store\_covariance = False$, $tol = 0.0001$.

5.2 Regressor Settings

We have successfully and promisingly applied the random forest classifier as shown in the comparison displayed in Figure S4. Based on these results, we have decided to further test a random forest regressor, using the parameters and results summarized in Figure 3 of the main paper. For this task, we have used the scikit-learn ver. 1.3.0 library's random forest regressor (Pedregosa et al., 2011), with its default parameters.

- **Random forest regressor (RFR):** $n\_estimators = 200$, $criterion = squared\_error$, $max\_depth = None$, $min\_samples\_split = 2$, $min\_samples\_leaf = 1$, $min\_weight\_fraction\_leaf = 0.0$, $max\_features = 1.0$, $max\_leaf\_nodes = None$, $min\_impurity\_decrease = 0.0$, $bootstrap = True$, $oob\_score = False$, $n\_jobs = None$, $random\_state = None$, $verbose = 0$, $warm\_start = False$, $ccp\_alpha = 0.0$, $max\_samples = None$.

6 ADDITIONAL UNSUPERVISED CLUSTERING RESULTS USING T-SNE

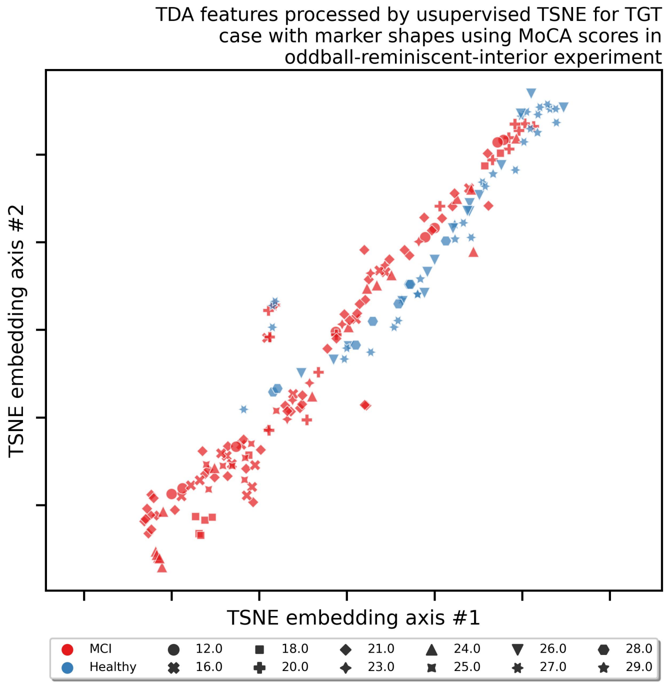

(a) TSNE of targets (TGT)

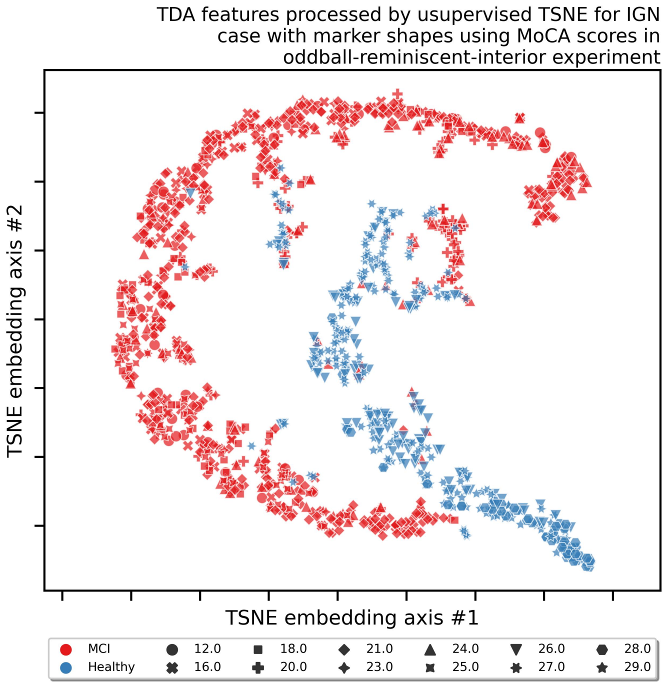

(b) TSNE of ignored (IGN)

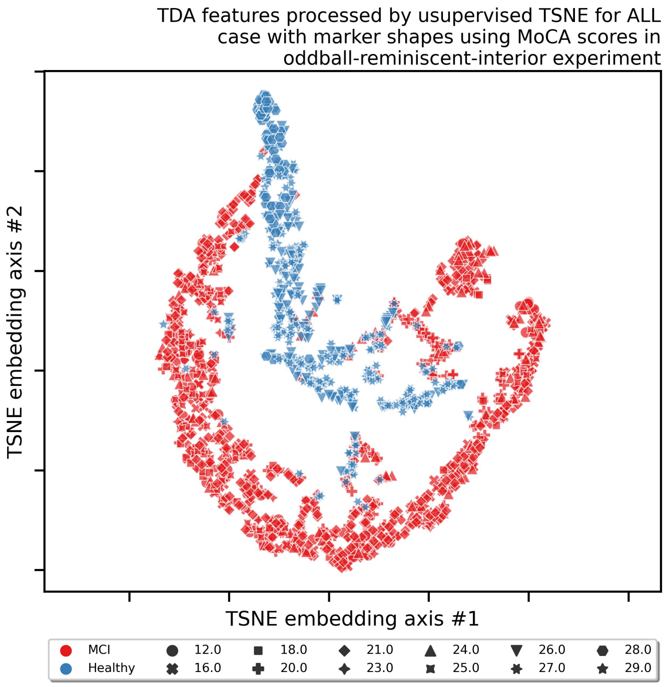

(c) TSNE of all (ALL)

Figure S5. Results of an unsupervised clustering t-SNE (Van der Maaten and Hinton, 2008) (a machine learning training without class labels) in form of scatter plots in three experimental response conditions (TGT: targets; IGN: ignored; and ALL: all responses together). Colors mark MCI (red) vs. normal aging cognition (blue), and marker shapes depict actual MoCA scores.

REFERENCES

Bauer, U. (2021). Ripser: efficient computation of Vietoris-Rips persistence barcodes. *J. Appl. Comput. Topol.* 5, 391–423. doi:10.1007/s41468-021-00071-5

[Dataset] Cycling '74 (2019). Max 8

Donchin, E. and Coles, M. G. (1988). Is the P300 component a manifestation of context updating. *Behavioral and brain sciences* 11, 357–427

Huang, N., Shen, Z., Long, S., Wu, M., Shih, H., Zheng, Q., et al. (1998). The empirical mode decomposition and the Hilbert spectrum for nonlinear and non-stationary time series analysis. *Proceedings of the Royal Society A: Mathematical, Physical and Engineering Sciences* 454, 903–995

[Dataset] Laszuk, D. (2017). Python implementation of empirical mode decomposition algorithm. `https://github.com/laszukdawid/PyEMD`. doi:10.5281/zenodo.5459184

Pedregosa, F., Varoquaux, G., Gramfort, A., Michel, V., Thirion, B., Grisel, O., et al. (2011). Scikit-learn: Machine learning in Python. *Journal of Machine Learning Research* 12, 2825–2830

Rutkowski, T. M., Abe, M. S., Komendzinski, T., and Otake-Matsuura, M. (2021). Older adult mild cognitive impairment prediction from multiscale entropy EEG patterns in reminiscent interior image working memory paradigm. In *2021 43rd Annual International Conference of the IEEE Engineering in Medicine Biology Society (EMBC)*. 6345–6348. doi:10.1109/EMBC46164.2021.9629480

Rutkowski, T. M., Abe, M. S., Komendzinski, T., Sugimoto, H., Narebski, S., and Otake-Matsuura, M. (2023). Machine learning approach for early onset dementia neurobiomarker using eeg network topology features. *Frontiers in Human Neuroscience* 17. doi:10.3389/fnhum.2023.1155194

Rutkowski, T. M., Abe, M. S., Tokunaga, S., Sugimoto, H., Komendziński, T., and Otake-Matsuura, M. (2022). Passive BCI oddball paradigm for dementia digital neuro-biomarker elucidation from attended and inhibited ERPs utilizing information geometry classification approaches. In *2022 IEEE International Conference on Systems, Man, and Cybernetics (SMC)* (IEEE Press), 2657–2662. doi:10.1109/SMC53654.2022.9945159

Rutkowski, T. M., Cichocki, A., and Mandic, D. (2008a). Information fusion for perceptual feedback: A brain activity sonification approach. In *Signal Processing Techniques for Knowledge Extraction and Information Fusion*, eds. D. Mandic, M. Golz, A. Kuh, D. Obradovic, and T. Tanaka (Springer US). 261–273

Rutkowski, T. M., Cichocki, A., Tanaka, T., Ralescu, A. L., and Mandic, D. P. (2008b). Clustering of spectral patterns based on emd components of eeg channels with applications to neurophysiological signals separation. In *Advances in Neuro-Information Processing* (Springer). 453–460

Rutkowski, T. M., Mandic, D. P., Cichocki, A., and Przybyszewski, A. W. (2010). EMD approach to multichannel EEG data - the amplitude and phase components clustering analysis. *Journal of Circuits, Systems, and Computers (JCSC)* 19, 215–229. doi:DOI:10.1142/S0218126610006037

Rutkowski, T. M. and Mori, H. (2015). Tactile and bone-conduction auditory brain computer interface for vision and hearing impaired users. *Journal of Neuroscience Methods* 244, 45 – 51. doi:10.1016/j.jneumeth.2014.04.010

Rutkowski, T. M., Toshihisa, T., Cichocki, A., and Mandic, D. P. (2008c). Clustering EMD components for muscular interference separation from EEG - a time/frequency approach with different distance measures. In *Proceedings of 23rd SIP Symposium* (Kanazawa: IEICE), 52–57

Schomer, D. L. and Lopes da Silva, F. H. (eds.) (2011). *Niedermeyer's Electroencephalography: Basic Principles, Clinical Applications, and Related Fields* (Wolters & Kluwer - Lippincott Williams & Wilkins), 6 edn.

Tralie, C., Saul, N., and Bar-On, R. (2018). Ripser.py: A lean persistent homology library for python. *The Journal of Open Source Software* 3, 925. doi:10.21105/joss.00925

Van der Maaten, L. and Hinton, G. (2008). Visualizing data using t-SNE. *Journal of machine learning research* 9

Varley, T. F., Denny, V., Sporns, O., and Patania, A. (2021). Topological analysis of differential effects of ketamine and propofol anaesthesia on brain dynamics. *Royal Society open science* 8, 201971

Wolpaw, J. and Wolpaw, E. W. (eds.) (2012). *Brain-Computer Interfaces: Principles and Practice* (New York, USA: Oxford University Press)
